# Increasing the Efficiency of a Control System for Detecting the Type and Amount of Oil Product Passing through Pipelines Based on Gamma-Ray Attenuation, Time Domain Feature Extraction, and Artificial Neural Networks

**DOI:** 10.3390/polym14142852

**Published:** 2022-07-13

**Authors:** Abdulilah Mohammad Mayet, Seyed Mehdi Alizadeh, Zana Azeez Kakarash, Ali Awadh Al-Qahtani, Abdullah K. Alanazi, John William Grimaldo Guerrero, Hala H. Alhashimi, Ehsan Eftekhari-Zadeh

**Affiliations:** 1 Electrical Engineering Department, King Khalid University, P.O. Box 394, Abha 61411, Saudi Arabia; amayet@kku.edu.sa (A.M.M.); alawalqahtani@kku.edu.sa (A.A.A.-Q.); 2 Petroleum Engineering Department, Australian College of Kuwait, West Mishref 13015, Kuwait; s.alizadeh@ack.edu.kw; 3 Department of Computer Science, Kurdistan Technical Institute, Sulaymaniyah 46001, Iraq; zana.azeez@kti.edu.krd; 4 Department of Engineering, Faculty of Engineering and Computer Science, Qaiwan International University, Sulaymaniyah 46001, Iraq; 5 Department of Chemistry, Faculty of Science, Taif University, P.O. Box 11099, Taif 21944, Saudi Arabia; aalanaz4@tu.edu.sa; 6 Department of Energy, Universidad de la Costa, Barranquilla 080001, Colombia; 7 Department of Physics, College of Science, Imam Abdulrahman Bin Faisal University, P.O. Box 1982, Dammam 31441, Saudi Arabia; halhashim@iau.edu.sa; 8 Institute of Optics and Quantum Electronics, Friedrich Schiller University Jena, Max-Wien-Platz 1, 07743 Jena, Germany

**Keywords:** detection system, feature extraction, RBF neural network, oil and polymeric fluids, dual-energy gamma source

## Abstract

Instantaneously determining the type and amount of oil product passing through pipelines is one of the most critical operations in the oil, polymer and petrochemical industries. In this research, a detection system is proposed in order to monitor oil pipelines. The system uses a dual-energy gamma source of americium-241 and barium-133, a test pipe, and a NaI detector. This structure is implemented in the Monte Carlo N Particle (MCNP) code. It should be noted that the results of this simulation have been validated with a laboratory structure. In the test pipe, four oil products—ethylene glycol, crude oil, gasoil, and gasoline—were simulated two by two at various volume percentages. After receiving the signal from the detector, the feature extraction operation was started in order to provide suitable inputs for training the neural network. Four time characteristics—variance, fourth order moment, skewness, and kurtosis—were extracted from the received signal and used as the inputs of four Radial Basis Function (RBF) neural networks. The implemented neural networks were able to predict the volume ratio of each product with great accuracy. High accuracy, low cost in implementing the proposed system, and lower computational cost than previous detection methods are among the advantages of this research that increases its applicability in the oil industry. It is worth mentioning that although the presented system in this study is for monitoring of petroleum fluids, it can be easily used for other types of fluids such as polymeric fluids.

## 1. Introduction

In the oil industry, a pipe is usually used to transport various oil products, which significantly reduces the cost of product transfer operations. When one product is inside the pipe, and the next product is loaded into the pipe, the two products mix at a cross-section, which is called the interface region. Therefore, the implementation of a control system to determine the amount and type of product inside the pipe is very important. Previous researchers have used gamma-ray-based systems as a reliable system for determining the parameters of two-phase [1,2,3] and three-phase [4,5,6] flows. In [1], researchers used wavelet transform as an effective method for feature extraction. The structure consisted of a Cs-137 source, two sodium iodide detectors, and a test pipe. Three homogeneous, stratified, and annular flow regimes at different volume percentages were simulated using the MCNP code. All flow regimes were fully detectable and volumetric percentages were predicted with relatively high accuracy. Nazemi et al. [2] implemented a laboratory structure in which they implemented the three flow regimes of annular, stratified, and bubbly in a test pipe at different volume percentages. Two NaI detectors and a cesium source were placed on either side of the pipe. The received signals were applied to the RBF neural network without any characteristic extraction to determine the volume fraction and type of flow regimes. Roshani et al. used a sodium iodide detector to optimize the structure of the detection system [3]. Co-60 was utilized as the gamma source and the properties of count under full energy peaks of 1.173 and 1.333 MeV, and count under Compton continuum were extracted from the signals received by the detector. Due to the extraction of inappropriate features in this study, flow regimes were not fully classifiable. In [4], the researchers simulated a three-phase flow regime, including water, oil, and gas, only in an annular flow regime and at different volume percentages. In this study, an adaptive neuro-fuzzy inference system (ANFIS) has been used to predict volume percentages, which obtained relatively good accuracy for predicting volume percentages. Studies [5,6] examined the performance of a GMDH neural network and Jaya algorithm to determine volume percentages of three-phase flows, respectively. In Articles [7,8], Sattari et al. used time-domain feature extraction techniques to increase the accuracy of determining the type of flow regimes and volume percentages, and they implemented neural networks such as GMDH and MLP with the time characteristics. In previous studies [9], Roshani et al. examined the characteristics of count under Compton continuum, count under photopeak of 1.173 MeV, count under photopeak of 1.333 MeV, and average value. By using a GMDH neural network, they recognized the type of flow regime and the volume percentage of two-phase flows with high accuracy. In another study [10], the thickness of the scale inside the pipe was predicted using the characteristics of counts under photopeak of Ba-133 and Cs-137 with energy of 356 keV and 662 keV and the RBF neural network. In [11], the researchers implemented a structure to control the type and amount of petroleum product in the pipe, which consisted of a dual-energy gamma source, a test pipe, and a detector. Although the amount and type of volume percentages were detectable in this study, the lack of use of feature extraction techniques prevents the achievement of a high-precision system.

In several previous studies, X-ray tubes have been used more than radioisotopes due to the ability to turn them off; this advantage limits potential health risks for people working with this device. In studies [12,13], the authors used a structure based on an X-ray tube to detect the type of flow regime and the volume percentages of multiphase flows. In [12], time characteristics, namely variance, skewness, kurtosis, Summation of Square Roots (SSR), and Summation of Variable Exponent Roots (SVER), were extracted from the received signal from a detector and defined as inputs for the MLP neural network. In [13], a three-phase flow was investigated. An X-ray tube, a pipe, and two NaI detectors were used in the structure of the detection system. The received signals from the two detectors were transmitted to the frequency domain using Fast Fourier Transform (FFT) and frequency characteristics were extracted. Three RBF neural networks were trained with the tasks of determining volume percentages and detecting the type of flow regimes using frequency characteristics. In [14], X-ray tubes were used to implement a diagnostic system, but the lack of characteristic extraction techniques was a disadvantage of this system. In another study [15], wavelet transform characteristics were applied to develop previous work ([14]). In [15], the characteristics of the fifth stage approximation and the details of the first to fifth stages were extracted using wavelet transform and introduced as the inputs for the MLP neural network. In [16], in order to design a system for detecting the type and amount of petroleum products in a pipe, different time characteristics were extracted. By calculating the correlation of these characteristics with each other, the characteristics that have the least similarity with each other were considered as the input for the MLP neural network. In all previous research, gamma-based systems have been introduced as the golden standard and a reliable system for determining the parameters of multiphase flows.

In this research, following the implementation of a precise system to control the type and amount of product in the pipe, a dual-energy gamma-ray-based system with a detector and a test pipe is proposed. Extracting the characteristics of the received signals and implementing the RBF neural networks using these characteristics is the difference between the proposed system and previous research, which has significantly increased the accuracy of the detection system. The present paper is divided as follows: In Section 2, the simulated structure with the MCNP is reported. Section 3 describes the extracted features in detail. The next section contains descriptions of the RBF neural network. Section 5 and Section 6 are result and discussion and conclusion sections, respectively.

## 2. Simulation Geometry

**Figure 1:**
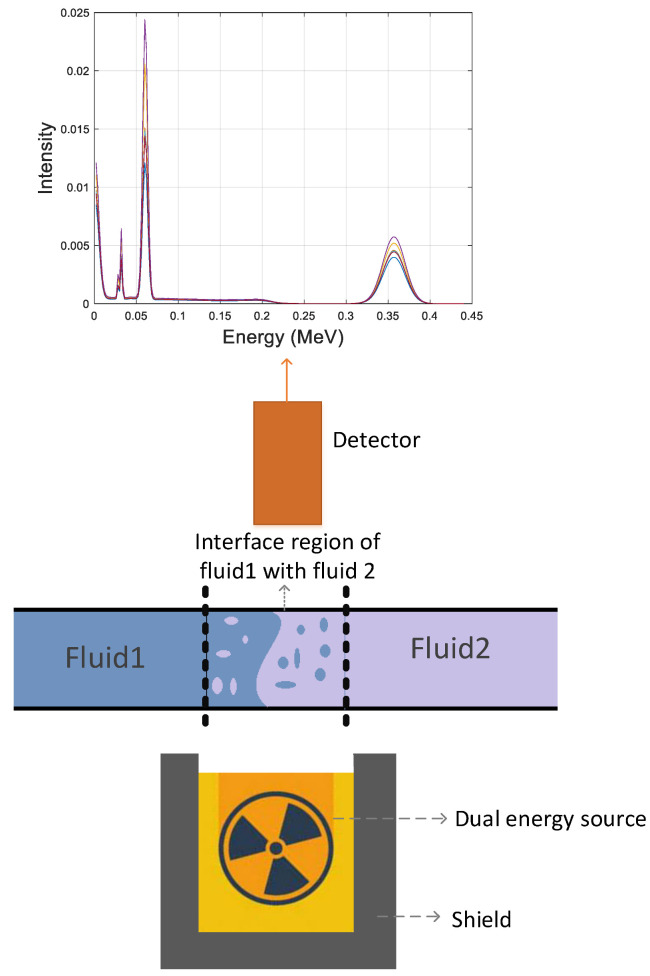
Simulated structure and sample signal recorded by the detector.

The purpose of this study is to propose a control system to determine the amount and type of oil product passing through pipelines. For this purpose, a test pipe with a dual-energy gamma source and a NaI detector on both sides is simulated. Ethylene glycol, crude oil, gasoline, and gasoil are the four petroleum products that have been studied in this study, and their density values are 1.114, 0.975, 0.721, and 0.826 g/cm^3^, respectively. This simulation was performed using a Monte Carlo N Particle (MCNP) code. When there is one product in the pipe and the other product is loaded in the pipe, in a cross-section, these two products are mixed. Subsequently, as the flow passes through the pipe, the amount of the first product decreases and the amount of the second product increases. The mentioned products were examined two by two in six different cases. All six possible modes were simulated at different volume ratios (from 5% to 95%). A total of 118 (6 different modes × 19 different volume ratios + 4 modes with only one product in the pipe) modes were simulated. The gamma source on one side of the pipe contains an americium-241 and barium-133. After the gamma-ray is emitted and collides with the test pipe and the products inside the pipe, the transmitted photons are collected by the detector, located on the other side of the pipe and directly in front of the source. The size of this detector is 25.4 mm × 25.4 mm and it is situated at a distance of 30 cm from the source. The simulated structure and sample of the signal received by the detector are shown in Figure 1. The simulation results of this study have been validated by previous studies [17]. In this study, several laboratory structures were implemented and compared to the results obtained from the MCNP code. Since the tally output in the MCNP code is per source particle, both were normalized to units to compare experimental and simulation data. The maximum relative error of 2.2% was the difference between the simulation results and the laboratory structure. The attenuation for the narrow gamma-ray beam follows Lambert–Beer’s law according to the following equation:

(1) $$I=I_{0}e^{-\mu \rho x}$$

where the intensity of un-collided and primary photons is shown by I and I_0_, respectively. µ and ρ represent the mass attenuation coefficient and density of absorber material, respectively. x is the beam path length through the absorber. According to this equation, different intensities are recorded by the detector due to the collision of photons with different objects. This difference in recorded intensity can be an important factor in determining the volume ratio of the petroleum product in the pipe.

## 3. Characteristic Extraction

The raw signals received from the detector need to be analyzed and processed so that they can be used as suitable inputs for the design of neural networks. To better interpret the received signals, reduce the size of the data, and reduce the computational costs imposed on neural networks, the feature extraction technique in the time domain was applied. Four time characteristics—variance, fourth order moment, skewness, and kurtosis—were extracted from the received signals with the following equations. The extracted features have been introduced as very useful features in previous research [7,8,18]. For this reason, in this research, in order to increase the efficiency of the control system, these characteristics have been extracted from the signals received by the detector.

- variance:

(2) $$m=\frac{1}{N}\sum_{n=1}^{N}x_{n}$$

(3) $${\;\sigma }^{2}=\frac{1}{N}\sum_{n=1}^{N}{\left(x_{n}-m\right)}^{2}$$

- fourth order moment:

(4) $$m_{4}=\frac{1}{N}\sum_{n=1}^{N}{\left[x_{n}-m\right]}^{4}$$

- skewness:

(5) $$\mathrm{Skewness}=\frac{m_{3}}{\sigma^{3}},{\;m}_{3}=\frac{1}{N}\sum_{n=1}^{N}{\left[x_{n}-m\right]}^{3}$$

- kurtosis:

(6) $$\mathrm{Kurtosis}=\frac{m_{4}}{\sigma^{4}}$$

where n is the number of data sets, N is the total number of data, and X_n_ denotes main signal in time domain.

## 4. Radial Basis Function Neural Network

In recent years, different mathematical approaches have been used for analyzing data in plenty of engineering fields [19,20,21,22,23,24,25,26,27,28,29,30,31,32,33,34,35,36,37,38,39,40,41,42,43,44,45,46], but it has been proven that an artificial neural network (ANN) is the most well-known and powerful tool for prediction and classification. RBF neural networks, abbreviated as “Radial Basis Function”, are special types of artificial neural networks that are distance-based and measure the similarity between data based on distance. An RBF network is a type of feed-forward artificial neural network that consists of three layers: the input layer, the hidden layer, and the output layer. Hidden layer neurons are activated by a Radial Basis Function. The most common form of radial base function is as follows [47]:

(7) $$\varphi \left(r\right)=\exp\left[-\frac{r^{2}}{2\sigma^{2}}\right]$$

r is the numerical value of the distance from the center of the cluster. Equation (7) shows a normal bell-shaped curve. A hidden layer consists of an array of computational units called hidden nodes. Each hidden node contains a central vector c, which is a parametric vector of length similar to the input vector x. The Euclidean distance between the center vector and the input vector x of the network is defined as [48]:

(8) $$r_{j}=\sqrt{\sum_{i=1}^{n}{\left(x_{i}-w_{\mathrm{ij}}\right)}^{2}}$$

Therefore, the output of the jth neuron in the hidden layer is as follows:

(9) $$\emptyset_{j}=\exp\left[-\frac{\sum_{i=1}^{n}{\left(x_{i}-w_{\mathrm{ij}}\right)}^{2}}{2\sigma^{2}}\right]$$

The width or radius of the bell curve is described by σ. The hidden layer of an RBF network has units that are weighted, and these weights corresponding to the vector represent the center of the cluster. Weights can be obtained using traditional methods such as the K-Mean algorithm or techniques based on the Kohonen algorithm. In any case, the training is performed non-supervised but the number of expected clusters (k) is pre-selected, these algorithms then obtain the best fit for these clusters. To design a neural network, the available data are usually divided into two categories: training and testing data. Training data contains more data (usually 70%). With this data, the neural network is implemented and the final model is fitted to the data. After this step, the function of the neural network should be tested. For this purpose, test data is given as input to the network. It should be noted that the neural network has not seen this data before, and the correct response to this data can assure that the network is functioning properly. MATLAB (2016, The MathWorks Inc., Natick, MA, USA) software was used to extract the characteristics mentioned in the previous section and to implement the RBF neural network. In this MATLAB software, there are several toolboxes for implementing neural networks; however, in this research, no pre-designed toolboxes have been used to implement neural networks and all stages of training and testing the neural networks have been programmed step-by-step for more freedom of action. It should be noted that the “newrb” function has been used to train the neural network. After providing the appropriate inputs, the neural network design operation began. The goal was to determine the volume ratio of the four oil products passing through the pipeline, which are mixed in a cross-section. For this purpose, four neural networks with the same inputs were designed, in which the output of each neural network is a percentage of the volume ratio of each product. There are various methods to show the function of neural networks but one of the most widely used methods is the use of regression and error diagrams. To increase productivity and increase speed in neural network training, available data should be optimized. There are various optimization methods that can be used according to the type and nature of available data [49,50,51,52,53]. In this research, in order to optimize the data, all the input and output data were transferred to a range between 0 and 1, and after training the network, the output data were returned to their original state.

## 5. Result and Discussion

**Figure 2:**
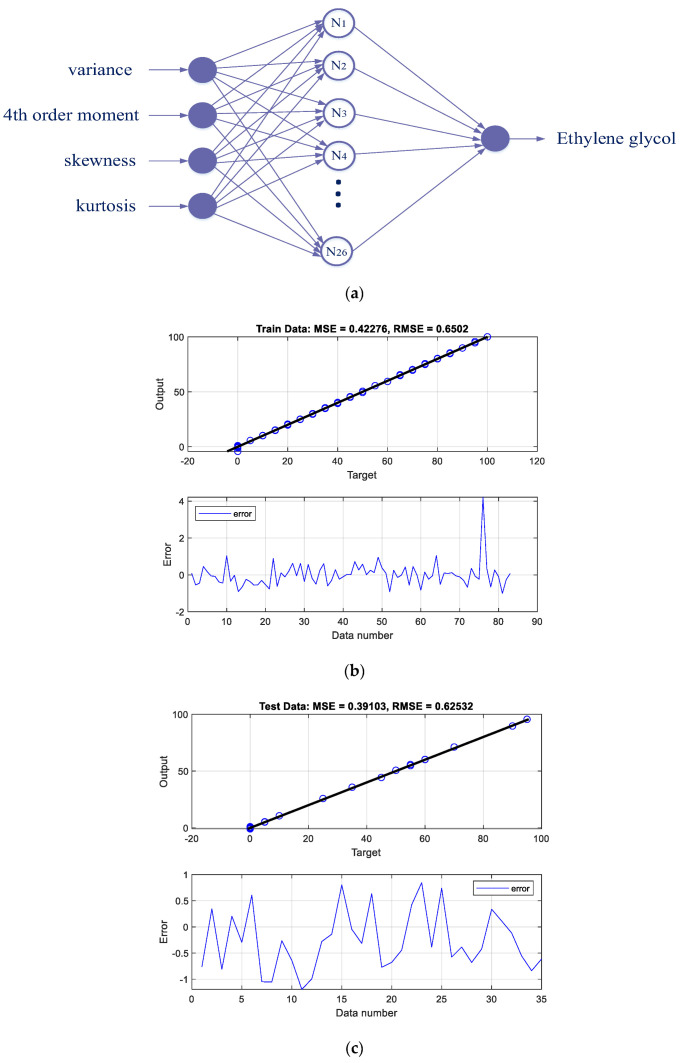
(**a**) The structure of the ethylene glycol volume ratio predictor network, (**b**) network performance against training, and (**c**) testing data.

**Figure 3:**
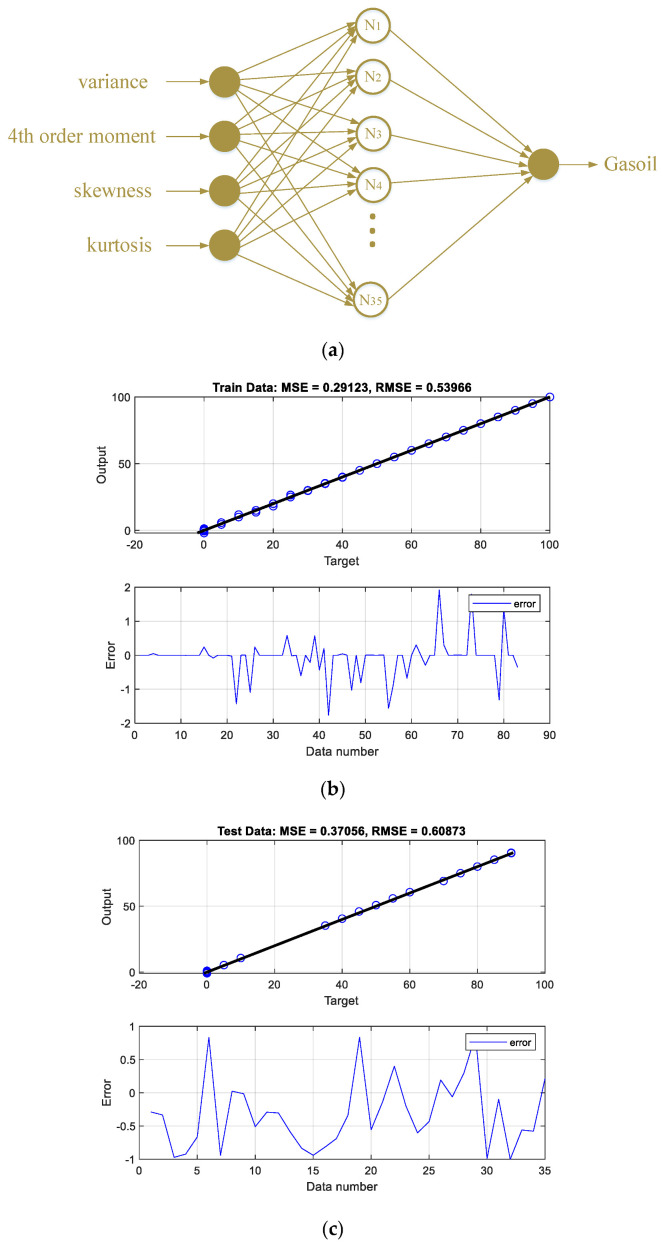
(**a**) The structure of the gasoil volume ratio predictor network, (**b**) network performance against training, and (**c**) testing data.

**Figure 4:**
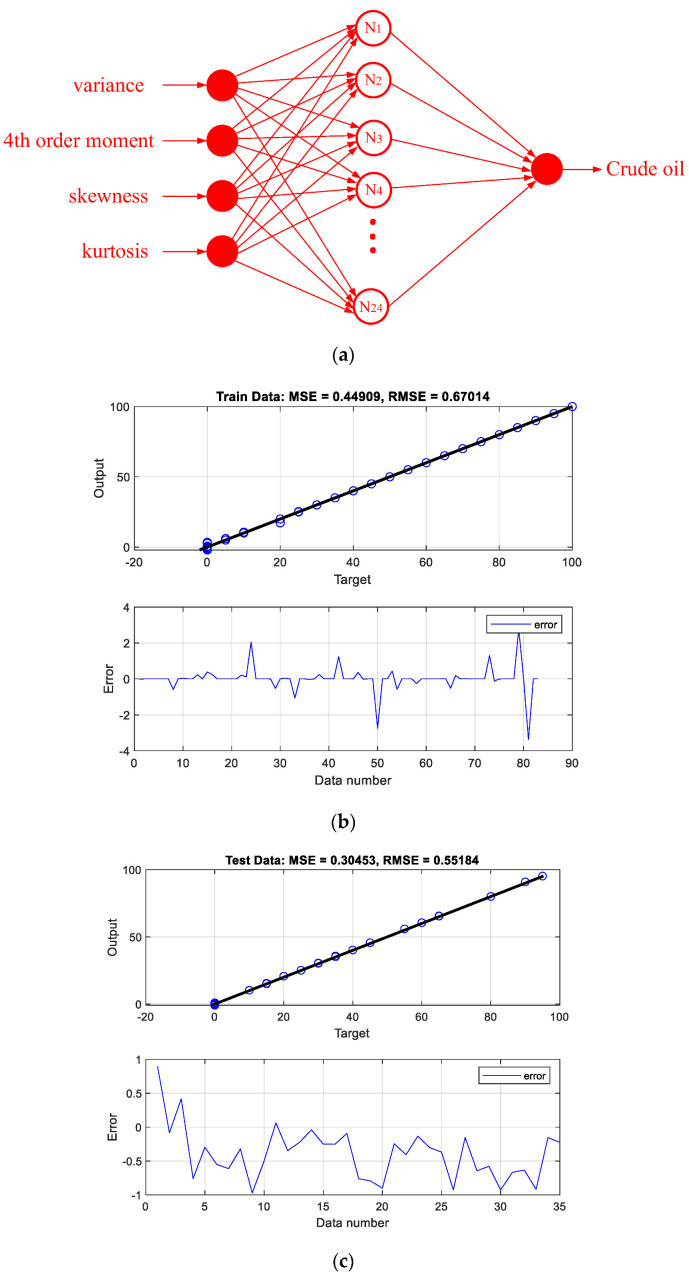
(**a**) The structure of the crude oil volume ratio predictor network, (**b**) network performance against training, and (**c**) testing data.

**Figure 5:**
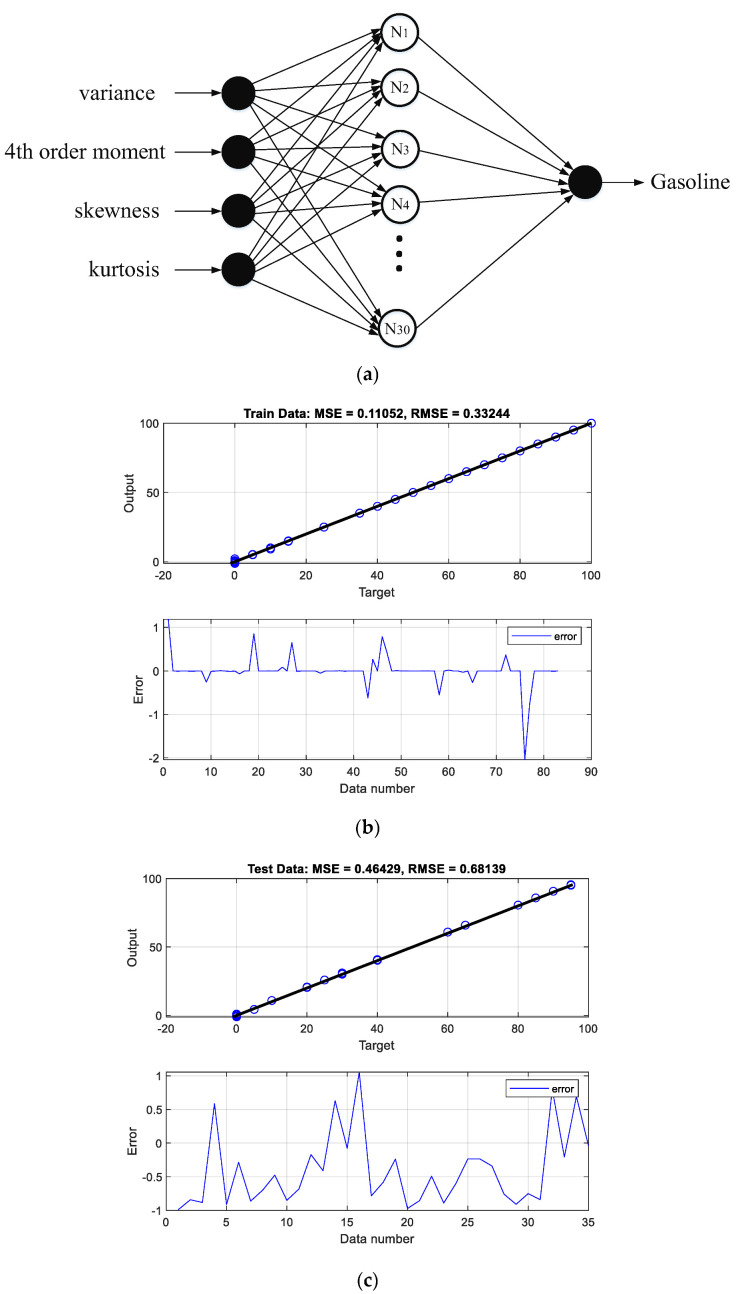
(**a**) The structure of the gasoline volume ratio predictor network, (**b**) network performance against training, and (**c**) testing data.

**Figure 6:**
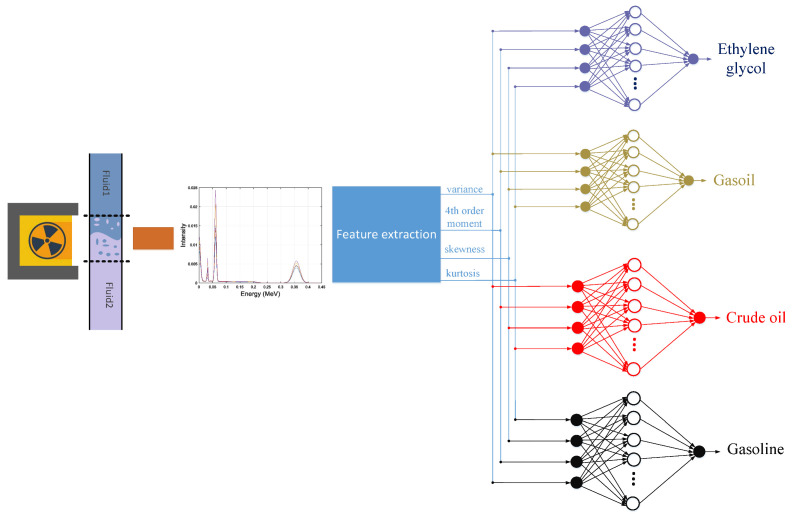
The general process of determining the type and amount of petroleum products.

The performance of the designed networks can be seen in the regression and error diagrams for the training and test data shown in Figure 2, Figure 3, Figure 4 and Figure 5. In the regression diagrams, the black line represents the desired outputs and the blue circle represents the output of the neural network. The error diagram shows the amount of error between the target data and the network output for each sample. The greater compatibility of the line and the circle indicates the high accuracy of the designed network. The specifications of these networks are shown in Table 1. The general process of the proposed detection system can be seen in Figure 6. The general process of the current research is that four oil products mixed two by two at different volume rates were first simulated in a test pipe. On both sides of this pipe, a dual-energy gamma source and a sodium iodide detector were placed. The detector provides the intensity of the transmitted photons. Four characteristics—variance, fourth order moment, skewness, and kurtosis—were extracted from the received spectrum to reduce dimensions, allow for better interpretation of data, and reduce computational load. The extracted features were considered as the inputs of four RBF neural networks. Each of these neural networks is responsible for determining the volume percentages of a product. By operating these four neural networks simultaneously, the type of product and volume percentage in the pipe can be recognized. One of the advantages of this research is reducing computational load. In this way, by manually extracting the characteristic, it has become easier for the neural network to assign the data and predict the volume rates with a fewer number of neurons.

In order to calculate the error of the designed neural networks, two error criteria (mean square error (MSE) and root mean square error (RMSE)) were calculated with the following equations, and the obtained values are shown in Table 1. Proper feature extraction is always one of the most important steps in signal analysis. Failure to use feature extraction techniques or not selecting the appropriate feature to determine volume ratio can be an obstacle to achieving high efficiency regarding the detection system. As can be seen from Table 1, the calculated error of the designed networks is very low, which is due to the application of appropriate inputs to the neural networks. Appropriate inputs in this study have been obtained by extracting time characteristics. The use of these features as input to other neural networks to achieve higher efficiency is highly recommended in future research. The target outputs and the outputs of all four designed neural networks are shown in Table 2. The comparison of this detection system with the others is shown in Table 3. It is clear that, by extracting the convenient characteristics, the error of the system can be outstandingly decreased.

(10) $$\mathrm{MSE}=\frac{\sum_{j=1}^{N}{\left(X_{j}\left(\mathrm{Exp}\right)-X_{j}\left(\mathrm{Pred}\right)\right)}^{2}}{N}$$

(11) $$\mathrm{RMSE}={\left[\frac{\sum_{j=1}^{N}{\left(X_{j}\left(\mathrm{Exp}\right)-X_{j}\left(\mathrm{Pred}\right)\right)}^{2}}{N}\right]}^{0.5}$$

## 6. Conclusions

Designing an effective and reliable control system is very important in the oil industry. For this purpose, a structure consisting of a dual-energy gamma source and a NaI detector was simulated. It should be noted that a pipe has also been used to simulate different combinations of petroleum products. After simulating two by two combinations of four different oil products at different volume ratios, the received signals from the detector were processed. From each of the received signals, four time characteristics—variance, fourth order moment, skewness, and kurtosis—were extracted and defined as inputs of neural networks. Four RBF neural networks were designed to determine the amount of each oil product. The simultaneous operation of four neural networks allows the amount and type of product within the pipeline to be measured quickly. The maximum RMSE of designed networks is 0.68, which is a very low error value for predicting volume ratios. This low error has turned the system into an efficient system that can help the oil industry to control oil products crossing the pipeline. The use of this methodology to determine parameters, such as the type of flow regimes and volume percentages of multiphase flows, as well as the use of deep neural networks can be considered as future research topics. Due to being unable to turn off gamma sources, users who use these sources must wear protective clothing, which is a major limitation in this research.

## Figures and Tables

**Table 1:** Specifications of designed networks.

Output	Ethylene Glycol		Gasoil		Crude Oil		Gasoline	
**Goal of MSE**	0		0		0		0	
**RBF spread**	3		1		2		2	
**Number of neurons in hidden layer**	26		35		24		30	
**Calculated MSE**	Train data	Test data	Train data	Test data	Train data	Test data	Train data	Test data
	0.42	0.39	0.29	0.37	0.44	0.30	0.11	0.46
**Calculated RMSE**	0.65	0.62	0.53	0.60	0.67	0.55	0.33	0.68

**Table 2:** Comparison of target values with neural network outputs.

Ethylene Glycol				Gasoil				Crude Oil				Gasoline			
Train		Test		Train		Test		Train		Test		Train		Test	
Target	Output	Target	Output	Target	Output	Target	Output	Target	Output	Target	Output	Target	Output	Target	Output
0	−0.0799	55	55.7664	85	85.0000	0	0.2894	0	0.0222	0	−0.8937	0	−1.1869	30	30.9895
0	0.5451	90	89.6545	0	0.0002	5	5.3342	45	45.0002	80	80.0845	15	15.0000	25	25.8423
50	50.4474	0	0.8107	80	79.9998	0	0.9726	70	70.0003	0	−0.4189	0	0.0016	10	10.8828
0	−0.4549	55	54.7951	0	−0.0461	0	0.9229	0	0.0012	35	35.7593	85	84.9999	0	−0.5858
0	−0.1717	0	0.2951	0	0.0002	60	60.6648	0	−0.0001	0	0.2964	0	0.0001	60	60.9125
15	15.0589	45	44.3924	30	30.0000	70	69.1665	45	45.0013	60	60.5500	0	0.0045	0	0.2852
0	0.0763	0	1.0496	0	−0.0001	0	0.9414	0	−0.0001	65	65.6113	0	−0.0004	25	25.8621
85	85.3810	0	1.0521	20	20.0000	0	−0.0233	10	10.5947	30	30.3222	0	0.0001	90	90.6989
35	35.4435	0	0.2638	45	45.0000	0	0.0150	0	0.0011	0	0.9706	5	5.2539	0	0.4766
0	−1.0354	10	10.6388	35	35.0000	0	0.5105	0	−0.0382	15	15.5022	0	0.0069	0	0.8508
0	0.3498	70	71.1897	15	15.0025	90	90.2923	60	59.9990	0	−0.0634	0	−0.0000	90	90.6827
100	100.021	0	0.9943	95	95.0000	85	85.3037	70	70.0007	0	0.3461	15	14.9922	30	30.1708
0	0.9045	60	60.2795	45	45.0000	40	40.5852	0	−0.2289	40	40.2242	35	34.9999	20	20.4114
95	95.6545	60	60.1388	0	−0.0000	0	0.8348	50	49.9994	0	0.0400	0	0.0077	5	4.3704
20	20.2404	0	−0.8026	0	−0.2426	55	55.9411	0	−0.3832	0	0.2488	75	75.0001	30	30.0776
75	75.3576	0	0.0451	0	0.0000	50	50.8200	0	−0.2498	35	35.2515	35	35.0650	0	−1.0564
20	20.5529	0	0.3146	20	20.0823	10	10.6899	80	80.0000	0	0.0930	0	−0.0028	0	0.7847
55	55.5412	0	−0.6346	0	0.0000	35	35.3375	80	80.0000	20	20.7608	0	0.0004	80	80.5792
80	80.2990	35	35.7716	65	65.0000	0	−0.8344	0	−0.0000	0	0.7931	10	9.1463	0	0.2380
65	65.5295	50	50.6779	95	95.0000	0	0.5560	0	0.0001	0	0.9005	0	−0.0000	0	0.9723
0	0.7638	0	0.4435	0	0.0292	0	0.1305	5	4.9997	95	95.2443	0	0.0004	60	60.8552
0	−0.8924	0	−0.4237	25	26.4295	0	−0.3993	0	−0.2098	65	65.4068	0	−0.0006	95	95.4927
0	0.6232	0	−0.8455	0	0.0000	0	0.1989	0	−0.1053	15	15.1329	65	64.9998	65	65.8907
25	24.8888	5	5.3846	0	−0.0037	0	0.6041	0	−2.0528	10	10.3023	40	39.9988	20	20.6045
25	25.1076	0	−0.7401	0	1.0932	0	0.4331	90	90.0000	0	0.3670	0	−0.0886	40	40.2360
65	64.7951	0	0.5763	30	29.7598	0	−0.1917	95	95.0000	55	55.9250	60	59.9999	0	0.2360
0	−0.6268	0	0.3852	55	55.0011	75	75.0616	0	0.0012	15	15.1520	0	−0.6505	0	0.3405
0	0.0490	0	0.6798	0	0.0000	0	−0.2948	0	0.0024	45	45.6444	0	0.0059	20	20.7590
0	−0.6229	0	0.4220	0	0.0000	0	−0.8617	0	0.5337	30	30.5781	0	−0.0005	0	0.9098
0	0.3576	90	89.6623	90	90.0000	45	45.9891	0	−0.0071	90	90.9244	95	95.0000	40	40.7515
40	39.4357	0	−0.1131	0	0.0019	80	80.0983	20	19.9598	0	0.6658	35	35.0000	85	85.8415
10	10.1857	5	5.1151	75	75.0000	0	1.0005	35	35.0022	0	0.6338	75	75.0000	0	−0.8019
0	0.4982	95	95.5550	0	−0.5813	0	0.5604	5	6.0738	0	0.9165	0	0.0495	0	0.2068
85	84.7521	25	25.8380	0	0.0196	90	90.5783	85	84.9999	25	25.1540	0	0.0006	0	−0.6944
50	49.3888	0	0.6127	100	100.000	0	−0.2137	85	85.0001	0	0.2235	45	45.0003	95	95.0399
75	75.5998	-	-	0	0.6066	-	-	40	40.0326	-	-	0	−0.0001	-	-
40	40.3048	-	-	0	0.0000	-	-	75	75.0001	-	-	0	−0.0064	-	-
0	−0.2791	-	-	0	0.2134	-	-	0	−0.2452	-	-	0	0.0021	-	-
15	15.2345	-	-	5	4.4292	-	-	0	−0.0000	-	-	10	9.9998	-	-
0	0.0998	-	-	0	0.4372	-	-	0	−0.0046	-	-	65	65.0000	-	-
0	−0.0330	-	-	30	29.8013	-	-	70	69.9999	-	-	80	79.9997	-	-
0	−0.0174	-	-	10	11.7721	-	-	0	−1.2382	-	-	70	70.0000	-	-
0	−0.7205	-	-	50	50.0000	-	-	50	50.0001	-	-	0	0.6206	-	-
20	19.7326	-	-	60	60.0000	-	-	30	29.9999	-	-	15	14.7291	-	-
60	59.4201	-	-	0	−0.0388	-	-	50	50.0023	-	-	0	−0.0000	-	-
0	−0.0077	-	-	0	−0.0017	-	-	0	−0.3565	-	-	0	−0.7858	-	-
0	−0.2518	-	-	0	1.0294	-	-	0	0.0142	-	-	0	−0.4207	-	-
15	14.8732	-	-	80	80.0000	-	-	65	65.0003	-	-	55	55.0000	-	-
0	−0.9549	-	-	5	5.8091	-	-	0	−0.0003	-	-	0	−0.0084	-	-
30	29.6232	-	-	95	95.0000	-	-	0	2.7451	-	-	0	−0.0025	-	-
75	74.9045	-	-	50	49.9970	-	-	0	−0.0040	-	-	50	49.9994	-	-
0	0.9123	-	-	15	15.0002	-	-	75	75.0004	-	-	85	85.0000	-	-
30	29.7462	-	-	0	−0.0003	-	-	0	−0.4358	-	-	0	−0.0002	-	-
30	30.1388	-	-	65	64.9998	-	-	0	0.5822	-	-	50	50.0000	-	-
80	80.0138	-	-	25	26.5605	-	-	95	95.0000	-	-	0	−0.0035	-	-
95	94.5685	-	-	0	0.8941	-	-	55	55.0000	-	-	0	−0.0004	-	-
45	45.5548	-	-	0	0.0009	-	-	0	0.0002	-	-	0	−0.0000	-	-
0	−0.4510	-	-	0	0.0000	-	-	25	25.2575	-	-	0	0.5518	-	-
0	0.0060	-	-	0	0.6777	-	-	85	85.0000	-	-	80	80.0000	-	-
0	0.8263	-	-	10	9.9997	-	-	40	39.9999	-	-	0	−0.0211	-	-
35	34.8439	-	-	0	−0.3033	-	-	0	0.0013	-	-	90	90.0000	-	-
70	70.2326	-	-	75	75.0000	-	-	75	75.0000	-	-	0	0.0001	-	-
10	10.0451	-	-	35	35.2954	-	-	0	−0.0009	-	-	0	0.0280	-	-
0	−1.0487	-	-	40	40.0000	-	-	0	−0.0009	-	-	0	0.0000	-	-
0	0.5138	-	-	55	55.0000	-	-	0	0.5212	-	-	0	0.2641	-	-
0	−0.1034	-	-	0	−1.9185	-	-	0	−0.1818	-	-	100	100.0000	-	-
0	−0.0799	-	-	40	39.7019	-	-	25	24.9999	-	-	50	50.0002	-	-
90	89.8810	-	-	0	0.0017	-	-	5	4.9890	-	-	70	70.0000	-	-
45	45.0490	-	-	0	0.0000	-	-	0	0.0021	-	-	45	45.0004	-	-
85	85.1076	-	-	60	59.9987	-	-	100	100.0000	-	-	0	0.0012	-	-
65	65.2951	-	-	0	−0.0000	-	-	60	59.9999	-	-	25	24.9994	-	-
5	5.6701	-	-	0	0.0000	-	-	90	89.9995	-	-	0	−0.3680	-	-
0	−0.3612	-	-	20	18.2032	-	-	0	−1.3001	-	-	5	5.0004	-	-
0	0.0724	-	-	70	70.0000	-	-	0	0.1209	-	-	55	55.0008	-	-
0	0.2326	-	-	85	85.0000	-	-	0	0.0029	-	-	0	−0.0036	-	-
0	−4.2127	-	-	0	−0.0000	-	-	10	9.9997	-	-	0	2.0414	-	-
70	69.6701	-	-	65	65.0000	-	-	55	55.0000	-	-	0	0.7757	-	-
0	0.6545	-	-	70	70.0000	-	-	0	−0.0000	-	-	45	45.0000	-	-
0	−0.2674	-	-	0	1.3162	-	-	20	17.2149	-	-	75	75.0002	-	-
80	80.0920	-	-	15	13.6166	-	-	0	0.0003	-	-	55	55.0004	-	-
0	1.0060	-	-	0	0.0000	-	-	0	3.3660	-	-	0	−0.0002	-	-
0	0.2482	-	-	25	25.0000	-	-	0	−0.0013	-	-	0	0.0022	-	-
40	39.9279	-	-	0	0.3553	-	-	0	−0.0004	-	-	70	70.0000	-	-

**Table 3:** A comparison of the error of the proposed detection system and previous studies.

Refs.	Extracted Features	Type of Neural Network	Maximum MSE	Maximum RMSE
**[7]**	Time-domain	GMDH	1.24	1.11
**[8]**	Time-domain	MLP	0.21	0.46
**[9]**	Lack of feature extraction	GMDH	7.34	2.71
**[54]**	Frequency-domain	MLP	0.67	0.82
**[55]**	Lack of feature extraction	MLP	17.05	4.13
**[56]**	Lack of feature extraction	MLP	2.56	1.6
**[current study]**	**Frequency-domain**	**RBF**	**0.46**	**0.68**

## Data Availability

Not applicable.

## References

[1] Hosseini S., Taylan O., Abusurrah M., Akilan T., Nazemi E., Eftekhari-Zadeh E., Bano F., Roshani G.H. (2021). Application of Wavelet Feature Extraction and Artificial Neural Networks for Improving the Performance of Gas–Liquid Two-Phase Flow Meters Used in Oil and Petrochemical Industries. Polymers.

[2] Nazemi E., Feghhi S.A.H., Roshani G.H., Peyvandi R.G., Setayeshi S. (2016). Precise Void Fraction Measurement in Two-phase Flows Independent of the Flow Regime Using Gamma-ray Attenuation. Nucl. Eng. Technol..

[3] Roshani G., Nazemi E., Feghhi S. (2016). Investigation of using 60 Co source and one detector for determining the flow regime and void fraction in gas–liquid two-phase flows. Flow Meas. Instrum..

[4] Roshani G.H., Karami A., Nazemi E., Shama F. (2018). Volume fraction determination of the annular three-phase flow of gas-oil-water using adaptive neuro-fuzzy inference system. Comput. Appl. Math..

[5] Roshani M., Phan G., Roshani G.H., Hanus R., Nazemi B., Corniani E., Nazemi E. (2021). Combination of X-ray tube and GMDH neural network as a nondestructive and potential technique for measuring characteristics of gas-oil–water three phase flows. Measurement.

[6] Roshani G.H., Karami A., Nazemi E. (2019). An intelligent integrated approach of Jaya optimization algorithm and neu-ro-fuzzy network to model the stratified three-phase flow of gas–oil–water. Comput. Appl. Math..

[7] Sattari M.A., Roshani G.H., Hanus R. (2020). Improving the structure of two-phase flow meter using feature extraction and GMDH neural network. Radiat. Phys. Chem..

[8] Sattari M.A., Roshani G.H., Hanus R., Nazemi E. (2021). Applicability of time-domain feature extraction methods and artificial intelligence in two-phase flow meters based on gamma-ray absorption technique. Measurement.

[9] Roshani M., Sattari M.A., Ali PJ M., Roshani G.H., Nazemi B., Corniani E., Nazemi E. (2020). Application of GMDH neural network technique to improve measuring precision of a simplified photon attenuation based two-phase flowmeter. Flow Meas. Instrum..

[10] Alamoudi M., Sattari M., Balubaid M., Eftekhari-Zadeh E., Nazemi E., Taylan O., Kalmoun E. (2021). Application of Gamma Attenuation Technique and Artificial Intelligence to Detect Scale Thickness in Pipelines in Which Two-Phase Flows with Different Flow Regimes and Void Fractions Exist. Symmetry.

[11] Roshani M., Phan G., Faraj R.H., Phan N.H., Roshani G.H., Nazemi B., Corniani E., Nazemi E. (2021). Proposing a gamma radia-tion based intelligent system for simultaneous analyzing and detecting type and amount of petroleum by-products. Nucl. Eng. Technol..

[12] Basahel A., Sattari M., Taylan O., Nazemi E. (2021). Application of Feature Extraction and Artificial Intelligence Techniques for Increasing the Accuracy of X-ray Radiation Based Two Phase Flow Meter. Mathematics.

[13] Taylan O., Sattari M.A., Essoussi I.E., Nazemi E. (2021). Frequency Domain Feature Extraction Investigation to Increase the Accuracy of an Intelligent Nondestructive System for Volume Fraction and Regime Determination of Gas-Water-Oil Three-Phase Flows. Mathematics.

[14] Roshani G.H., Ali P.J.M., Mohammed S., Hanus R., Abdulkareem L., Alanezi A.A., Sattari M.A., Amiri S., Nazemi E., Eftekhari-Zadeh E. (2021). Simula-tion Study of Utilizing X-ray Tube in Monitoring Systems of Liquid Petroleum Products. Processes.

[15] Balubaid M., Sattari M.A., Taylan O., Bakhsh A.A., Nazemi E. (2021). Applications of Discrete Wavelet Transform for Feature Extraction to Increase the Accuracy of Monitoring Systems of Liquid Petroleum Products. Mathematics.

[16] Mayet A.M., Alizadeh S.M., Nurgalieva K.S., Hanus R., Nazemi E., Narozhnyy I.M. (2022). Extraction of Time-Domain Characteristics and Selection of Effective Features Using Correlation Analysis to Increase the Accuracy of Petroleum Fluid Monitoring Systems. Energies.

[17] Nazemi E., Roshani G.H., Feghhi S.A.H., Setayeshi S., Zadeh E.E., Fatehi A. (2016). Optimization of a method for identifying the flow regime and measuring void fraction in a broad beam gamma-ray attenuation technique. Int. J. Hydrogen Energy.

[18] Sattari M.A., Korani N., Hanus R., Roshani G.H., Nazemi E. (2020). Improving the performance of gamma radiation based two phase flow meters using optimal time characteristics of the detector output signal extraction. J. Nucl. Sci. Technol..

[19] Isaev A.A., Aliev M.M.O., Drozdov A.N., Gorbyleva Y.A., Nurgalieva K.S. (2022). Improving the Efficiency of Curved Wells’ Operation by Means of Progressive Cavity Pumps. Energies.

[20] Lalbakhsh A., Mohamadpour G., Roshani S., Ami M., Roshani S., Sayem A.S.M., Alibakhshikenari M., Koziel S. (2021). Design of a Compact Planar Transmission Line for Miniaturized Rat-Race Coupler With Harmonics Suppression. IEEE Access.

[21] Roshani S., Roshani S. (2021). A compact coupler design using meandered line compact microstrip resonant cell (MLCMRC) and bended lines. Wirel. Netw..

[22] Shukla N.K., Mayet A.M., Vats A., Aggarwal M., Raja R.K., Verma R., Muqeet M.A. (2022). High speed integrated RF–VLC data communication system: Performance constraints and capac-ity considerations. Phys. Commun..

[23] Hookari M., Roshani S., Roshani S. (2020). High-efficiency balanced power amplifier using miniaturized harmonics suppressed coupler. Int. J. RF Microw. Comput.-Aided Eng..

[24] Mayet A., Hussain A., Hussain M. (2016). Three-terminal nanoelectromechanical switch based on tungsten nitride, an amor-phous metallic material. Nanotechnology.

[25] Lotfi S., Roshani S., Roshani S., Gilan M.S. (2020). Wilkinson power divider with band-pass filtering response and harmonics suppression using open and short stubs. Frequenz.

[26] Mayet A., Hussain M. Amorphous WNx Metal For Accelerometers and Gyroscope. Proceedings of the MRS Fall Meeting.

[27] Jamshidi M., Siahkamari H., Roshani S., Roshani S. (2019). A compact Gysel power divider design using U-shaped and T-shaped resonators with harmonics suppression. Electromagnetics.

[28] Mayet A., Smith C.E., Hussain M.M. Energy reversible switching from amorphous metal based nanoelectromechanical switch. Proceedings of the Nanotechnology (IEEE-NANO), 2013 13th IEEE Conference.

[29] Roshani S., Roshani S. (2017). Two-section impedance transformer design and modeling for power amplifier applications. Appl. Comput. Electromagn. Soc. J..

[30] Khaibullina K.S., Sagirova L.R., Sandyga M.S. (2020). Substantiation and selection of an inhibitor for preventing the formation of asphalt-resin-paraffin deposits. [Substanciação e seleção de um inibidor para evitar a formação de depósitos de asfalto-resina-parafina]. Period. Tche Quim..

[31] Jamshidi M.B., Roshani S., Talla J., Roshani S., Peroutka Z. (2021). Size reduction and performance improvement of a microstrip Wil-kinson power divider using a hybrid design technique. Sci. Rep..

[32] Mayet A., Smith C., Hussain M.M., Smith C. Amorphous metal based nanoelectromechanical switch. Proceedings of the 2013 Saudi International Electronics, Communications and Photonics Conference.

[33] Hookari M., Roshani S., Roshani S. (2020). Design of a low pass filter using rhombus-shaped resonators with an analytical LC equiv-alent circuit. Turk. J. Electr. Eng. Comput. Sci..

[34] Khaibullina K.S., Korobov G.Y., Lekomtsev A.V. (2020). Development of an asphalt-resin-paraffin deposits inhibitor and substantiation of the technological parameters of its injection into the bottom-hole formation zone. [Desenvolvimento de um inibidor de depósito de asfalto-resinaparafina e subscantiação dos parâmetros tecnológicos de sua injeção na zona de formação de furo inferior]. Period. Tche Quim..

[35] Pirasteh A., Roshani S., Roshani S. (2020). Design of a miniaturized class F power amplifier using capacitor loaded transmission lines. Frequenz.

[36] Tikhomirova E.A., Sagirova L.R., Khaibullina K.S. (2019). A review on methods of oil saturation modelling using IRAP RMS. IOP Conf. Ser. Earth Environ. Sci..

[37] Roshani S., Dehghani K., Roshani S. (2019). A lowpass filter design using curved and fountain shaped resonators. Frequenz.

[38] Khaibullina K. Technology to Remove Asphaltene, Resin and Paraffin Deposits in Wells Using Organic Solvents. Proceedings of the SPE Annual Technical Conference and Exhibition.

[39] Roshani S., Roshani S. (2020). Design of a compact LPF and a miniaturized Wilkinson power divider using aperiodic stubs with harmonic suppression for wireless applications. Wirel. Netw..

[40] Alanazi A.K., Alizadeh S.M., Nurgalieva K.S., Guerrero J.W.G., Abo-Dief H.M., Eftekhari-Zadeh E., Nazemi E., Narozhnyy I.M. (2021). Optimization of X-ray Tube Voltage to Improve the Precision of Two Phase Flow Meters Used in Petroleum Industry. Sustainability.

[41] Alanazi A.K., Alizadeh S.M., Nurgalieva K.S., Nesic S., Guerrero J.W.G., Abo-Dief H.M., Eftekhari-Zadeh E., Nazemi E., Narozhnyy I.M. (2022). Application of Neural Network and Time-Domain Feature Extraction Techniques for Determining Volumetric Percentages and the Type of Two Phase Flow Regimes Independent of Scale Layer Thickness. Appl. Sci..

[42] Mayet A.M., Salama A.S., Alizadeh S.M., Nesic S., Guerrero J.W.G., Eftekhari-Zadeh E., Nazemi E., Iliyasu A.M. (2022). Applying Data Mining and Artificial Intelligence Techniques for High Precision Measuring of the Two-Phase Flow’s Characteristics Independent of the Pipe’s Scale Layer. Electronics.

[43] Lalbakhsh A., Afzal M.U., Esselle K.P., Smith S.L. (2020). Low-Cost Non-Uniform Metallic Lattice for Rectifying Aperture Near-Field of Electromagnetic Bandgap Resonator Antennas. IEEE Trans. Antennas Propag..

[44] Paul G.S., Mandal K., Lalbakhsh A. (2021). Single-layer ultra-wide stop-band frequency selective surface using interconnected square rings. AEU-Int. J. Electron. Commun..

[45] Lalbakhsh A., Afzal M.U., Esselle K.P. Simulation-driven particle swarm optimization of spatial phase shifters. Proceedings of the 18th IEEE international Conference on Electromagnetics in Advanced Applications (ICEAA).

[46] Lalbakhsh A., Lotfi-Neyestanak A.A., Naser-Moghaddasi M. (2012). Microstrip Hairpin Bandpass Filter Using Modified Minkowski Fractal-for Suppression of Second Harmonic. IEICE Trans. Electron..

[47] Hartman E.J., Keeler J.D., Kowalski J.M. (1990). Layered neural networks with Gaussian hidden units as universal approximators. Neural Comput..

[48] Yu B., He X. Training radial basis function networks with differential evolution. Proceedings of the IEEE International Conference on Granular Computing.

[49] Çolak A.B. (2021). An experimental study on the comparative analysis of the effect of the number of data on the error rates of artificial neural networks. Int. J. Energy Res..

[50] Çolak A.B., Shafiq A., Sindhu T.N. (2022). Modeling of Darcy–Forchheimer bioconvective Powell Eyring nanofluid with artificial neural network. Chin. J. Phys..

[51] Shafiq A., Çolak A.B., Lone S.A., Sindhu T.N., Muhammad T. (2022). Reliability modeling and analysis of mixture of exponential distributions using artificial neural net-work. Math. Methods Appl. Sci..

[52] Hosseini S., Roshani G.H., Setayeshi S. (2020). Precise gamma based two-phase flow meter using frequency feature extraction and only one detector. Flow Meas. Instrum..

[53] Roshani M., Ali P.J., Roshani G.H., Nazemi B., Corniani E., Phan N.H., Tran H.N., Nazemi E. (2020). X-ray tube with artificial neural net-work model as a promising alternative for radioisotope source in radiation based two phase flowmeters. Appl. Radia-Tion Isot..

[54] Peyvandi R.G., Rad S.I. (2017). Application of artificial neural networks for the prediction of volume fraction using spectra of gamma rays backscattered by three-phase flows. Eur. Phys. J. Plus.

[55] Roshani Gholam H., Ehsan N., Farzin S., Imani Mohammad A., Salar M. (2018). Designing a simple radiometric system to predict void fraction percentage independent of flow pattern using radial basis function. Metrol. Meas. Syst..

[56] Roshani G.H., Nazemi E., Feghhi S.A.H., Setayeshi S. (2015). Flow regime identification and void fraction prediction in two-phase flows based on gamma ray attenuation. Measurement.

